# Kynurenic Acid Levels are Increased in the CSF of Alzheimer’s Disease Patients

**DOI:** 10.3390/biom10040571

**Published:** 2020-04-08

**Authors:** Marta González-Sánchez, Javier Jiménez, Arantzazu Narváez, Desiree Antequera, Sara Llamas-Velasco, Alejandro Herrero-San Martín, Jose Antonio Molina Arjona, Adolfo López de Munain, Alberto Lleó Bisa, M.-Pilar Marco, Montserrat Rodríguez-Núñez, David Andrés Pérez-Martínez, Alberto Villarejo-Galende, Fernando Bartolome, Elena Domínguez, Eva Carro

**Affiliations:** 1 Group of Neurodegenerative Diseases, Hospital 12 de Octubre Research Institute (imas12), 28041 Madrid, Spain; martags.86@gmail.com (M.G.-S.); eeara@yahoo.es (D.A.); laisset@hotmail.com (S.L.-V.); alexportalrubio@hotmail.com (A.H.-S.M.); cvillaiza@telefonica.net (J.A.M.A.); daperezm@salud.madrid.org (D.A.P.-M.); avgalende@yahoo.es (A.V.-G.); 2 Biomedical Research Networking Center in Neurodegenerative Diseases (CIBERNED), 28031 Madrid, Spain; adolfojose.lopezdemunainarregui@osakidetza.eus (A.L.d.M.); alleo@santpau.cat (A.L.B.); 3 Department of Analytical Chemistry, Physical Chemistry and Chemical Engineering, University of Alcalá, 28871 Alcalá de Henares, Spain; javier.jimenez@uah.es (J.J.); arantzazu.narvaez@uah.es (A.N.); 4 Neurosciences Research Area, Biodonostia Health Research Institute, 20014 San Sebastian, Spain; 5 Neurology Department, Hospital de la Santa Creu i Sant Pau, 08041 Barcelona, Spain; 6 Nanobiotechnology for Diagnostics (Nb4D) Group, Institute for Advanced Chemistry of Catalonia (IQAC), Spanish National Research Council (CSIC), 08034 Barcelona, Spain; pilar.marco@iqac.csic.es (M.-P.M.); montse.rodriguez@iqac.csic.es (M.R.-N.); 7 Centro de Investigación Biomédica en Red (CIBER) de Bioingeniería, Biomateriales y Nanomedicina (CIBER-BBN), 08034 Barcelona, Spain

**Keywords:** Alzheimer´s disease, kynurenine pathway, cerebrospinal fluid, biomarkers, amyloid-β, tau protein

## Abstract

Kynurenic acid (KYNA) is a product of the tryptophan (TRP) metabolism via the kynurenine pathway (KP). This pathway is activated in neurodegenerative disorders, such as Alzheimer´s disease (AD). KYNA is primarily produced by astrocytes and is considered neuroprotective. Thus, altered KYNA levels may suggest an inflammatory response. Very recently, significant increases in KYNA levels were reported in cerebrospinal fluid (CSF) from AD patients compared with normal controls. In this study, we assessed the accuracy of KYNA in CSF for the classification of patients with AD, cognitively healthy controls, and patients with a variety of other neurodegenerative diseases, including frontotemporal dementia (FTD), amyotrophic lateral sclerosis (ALS), and progressive supranuclear palsy (PSP). Averaged KYNA concentration in CSF was higher in patients with AD when compared with healthy subjects and with all the other differentially diagnosed groups. There were no significant differences in KYNA levels in CSF between any other neurodegenerative groups and controls. These results suggest a specific increase in KYNA concentration in CSF from AD patients not seen in other neurodegenerative diseases.

## 1. Introduction

Alzheimer’s disease (AD) is a complex and progressive neurodegenerative disorder that represents one of the major health problems in the world [1]. The main pathophysiological features of AD are the in-brain accumulation of amyloid-β peptide (Aβ) in neuritic extracellular plaques, the hyperphosphorylated tau protein in intracellular neurofibrillary tangles (NFT), and neuronal loss. The consequence of the brain accumulation of Aβ and tau can be detected by analyzing the levels of Aβ_42_ peptide (the most neurotoxic isoform of Aβ) and total tau (t-tau) protein in the cerebrospinal fluid (CSF) of AD patients. This analysis made it possible to establish these parameters as clinical biomarkers indicating that reduced Aβ levels and increased t-tau levels in CSF are distinctive of AD [2]. The inverse correlation between Aβ_42_ levels in the CSF and the brain is explained because Aβ_42_ in the AD brain is sequestered in the cerebral neuritic plaques, as it is their main component, with less Aβ being available to diffuse into the CSF, and therefore reduced levels of Aβ_42_ in CSF from AD patients (reviewed in [3]). In the case of t-tau protein, CSF level probably reflects the intensity of neuronal damage and neurodegeneration (reviewed in [3]). Although highly studied, the exact initiating and pathophysiological AD-initiating factors remain unknown, but the majority of AD-related research is based on the amyloid cascade and tau hypotheses. In addition to these theories, different pathological mechanisms, such as mitochondrial dysfunction, glutamate excitotoxicity, and neuroinflammation have been implicated in AD [2,3,4,5,6,7,8]. Neuroinflammation in AD is associated with the activation of microglia and astrocytes along with increased levels of neuroinflammatory mediators and modulators such as proinflammatory cytokines and chemokines [8,9]. One consequence of microglial activation is the overproduction of quinolinic acid (QA). This excitotoxin is a downstream-derived product from the kynurenine pathway (KP) and strongly contributes to the neuroinflammatory environment [10]. KP is the main route that metabolizes tryptophan (TRP), and particularly, this pathway has also been implicated in AD [11]. There is a bidirectional connection between inflammatory cytokines and the KP, and an imbalance in this association may possibly lead to neurological or psychiatric disorders [12,13]. One study suggested that interleukin (IL)-6 activates the KP in patients with schizophrenia, leading to an increased production of kynurenic acid (KYNA) [14]. It was shown that the KP regulates both innate and adaptive immune responses, and that TRP metabolism reflects a crucial interface between immune and central nervous system (CNS) [15]. Therefore, any imbalance in the KP and subsequently in the production of TRP metabolites may cause neuronal damage, giving rise to multiple physiological and neurological impairments [16,17].

TRP is an essential amino acid necessary for protein biosynthesis, and a precursor of several compounds with important biological functions, such as serotonin, melatonin, and kynurenines [18]. TRP enters the brain by a carrier-mediated transport across the blood-brain barrier, where it is lately degraded. KYNA is a product of the TRP metabolism via the KP, and it is synthesized in and released by astrocytes in the brain [19,20]. KYNA is an *N*-methyl-D-aspartate (NMDA) and α7 nicotinic acetylcholine (α7nACh) receptor antagonist and is considered to be neuroprotective [18,21,22,23]. It counteracts the excitotoxic activity of other KP metabolites, such as QA [24,25]. Increased levels of KYNA were found in AD brains and AD animal models, and authors explained that by exerting its antagonist abilities on NMDA and α7nACh receptors, KYNA may disrupt the learning and cognitive abilities [26,27]. Additionally, increased levels of KYNA in the developing brain resulted in biochemical impairments and behavioral abnormalities [28]. However, several reports showing KYNA and other KP metabolite levels in CSF and blood from AD patients and healthy subjects reached inconclusive results [29,30,31,32,33,34,35]. Using high-performance liquid chromatography (HPLC), recent studies found increased KYNA concentration in CSF in different cohorts of AD patients [36,37]. However, it was almost simultaneously published that KYNA levels were strongly reduced in CSF from AD patients when compared with control subjects [38]. In this work, we aimed to clarify KP metabolite concentrations measured in CSF and serum and to determine their specificity in AD. Therefore, we analyzed TRP and KYNA levels in CSF and plasma from a well-characterized cohort of patients who had mild cognitive impairment (MCI), AD, or one of a range of other neurodegenerative disorders, including frontotemporal dementia (FTD), amyotrophic lateral sclerosis (ALS), and progressive supranuclear palsy (PSP), using a sensitive enzyme-linked immunosorbent assay (ELISA).

## 2. Materials and Methods

### 2.1. Cohorts

In total, 132 subjects were included in this study: (i) elderly non-demented subjects classified as controls (*n* = 23); (ii) patients with mild cognitive impairment due to AD (MCI) (*n* = 24); (iii) probable mild AD (*n* = 41); (iv) moderate–severe AD (*n* = 20); (v) frontotemporal dementia (FTD) (*n* = 8); (vi) amyotrophic lateral sclerosis (ALS) (*n* = 8); and (vii) progressive supranuclear palsy (PSP) (*n* = 8). Subjects were recruited from the Neurology Service of three different geographical areas in Spain: 12 de Octubre University Hospital (Madrid); Donostia-Osakidetza University Hospital (San Sebastian); and Santa Creu i Sant Pau Hospital (Barcelona). Diagnoses were based on detailed clinical assessments, neuropsychological studies, neuroimaging, electromyography, and CSF Aβ_42_ and t-tau levels. An AD- indicative biomarker profile (Aβ^+^) was defined as CSF Aβ_42_ <550 pg/µL and t-tau/Aβ_42_ ratio >0.52, according to ELISA manufacture instructions and reported cut-offs [39]. Those cases with CSF Aβ_42_ ≥550 pg/µL and t-tau/Aβ_42_ ratio ≤0.52 were defined as non-AD biomarker profiles (Aβ^−^). Diagnosis of MCI and dementia due to AD were established according to the National Institute on Aging and the Alzheimer’s Association guidelines [2,40], and these criteria were used in the present study. The majority of MCI patients were amnesic MCI but some of them presented an atypical non-amnesic MCI (visual variant or language variant). These atypical cases were, however, then confirmed as MCI due to AD within their Aβ^+^ CSF biomarker profiles. Global cognition was assessed using the Mini Mental State Examination (MMSE) [41] and disease severity using the Clinical Dementia Rating (CDR) score [42]. All FTD patients met the clinical diagnosis criteria of behavioral variant FTD [43], or the nonfluent/semantic variant primary progressive aphasia (PPA) [44]. ALS and PSP diagnosis were established according to revised El Escorial criteria [45] and Boxer criteria [46] respectively. All MCI and AD dementia patients had an Aβ^+^ CSF biomarker profile, while it was Aβ^−^ in patients with other neurodegenerative diseases and healthy controls. Controls were usually recruited from among the patients’ partners when they fulfilled the age and health criteria. Inclusion criteria for old, cognitively-normal individuals were age over 50, no history or clinical signs of neurological or psychiatric disease, and absence of AD CSF biomarker profile. Exclusion criteria for patients were evidence of concomitant cerebrovascular disease or any neurological, psychiatric, or non-neurological medical conditions; or medications that could affect cognition or motor function. Consent was in accordance with the Declaration of Helsinki, and approval was obtained from the research ethics committee of the responsible institution (Research Institute Hospital 12 de Octubre (imas12: 16/079, Date: 26 April 2016); Biodonostia Health Research Institute (22/2016 Date: 19 July 2017); Sant Pau Biomedical Research Institute (16/2013 Date: 14 January 2014)). Written informed consent was signed by all participants or representatives.

### 2.2. Sample Collection

CSF samples were collected from all subjects and processed according to standardized procedures by lumbar puncture in 15-mL sterile polypropylene tubes. Samples were then centrifuged at 3000 rpm at 4 °C for 10 min. Supernatant aliquots were stored at −80 °C into 0.5 mL polypropylene tubes with Protease Inhibitor Cocktail (Roche Applied Science, Basel, Switzerland).

Blood samples were obtained through antecubital vein puncture from patients and healthy subjects. Plasma was isolated from whole blood and collected in 7-mL-EDTA-2Na tubes. Whole blood was centrifuged at 2000 rpm for 10 min at room temperature. Supernatants were then collected in tubes with Protease Inhibitor Cocktail (Roche Applied Science, Basel, Switzerland) and stored at −80 °C.

### 2.3. DNA Purification and Apolipoprotein E (APOE) Genotyping

Genomic DNA was extracted from peripheral blood using QIAmp DNA Blood Mini Kit (Qiagen, Hilden, Germany), according to the manufacturer instructions. Human *APOE* C112R and R158C polymorphisms were detected to identify the *APOE* ε2, ε3, and ε4 alleles, using LightCycler 480 II Instruments Kit (Roche Diagnostics, Basel, Switzerland) following manufacturer instructions.

### 2.4. Aβ_42_ and t-tau Quantification

Endogenous Aβ_42_ and t-tau levels in CSF samples were quantified using INNOTEST ELISAs (Fujirebio Europe NV, Gent, Belgium), according to manufacturer’s instructions.

### 2.5. KYNA and TRP Quantification

KYNA and 5-hydroxy-L-tryptophan (L-5-HTP; from now on TRP) levels in CSF and plasma samples were quantified using an in-house competitive ELISA in triplo developed by the Bioelectrochemistry and Biosensors Group from the University of Alcalá in collaboration with the Nanobiotechnology for Diagnostics (Nb4D) group from IQAC-CSIC. Analytical parameters found in CSF were a limit of detection (LOD) of 0.26 µg/L with an IC_50_ of 23.6 µg/L and a linear range (LR) from 2.8 to 100 µg/L for KYNA; and a LOD of 2.5 µg/L with an IC_50_ of 250 µg/L and a LR from 40–912 µg/L for TRP. The immunoreagents used for KYNA detection (the polyclonal antibody (As301) and the enzyme tracer (HRP-SIA-IHSH)) were developed by the Nb4D group with the support of the ICTS “NANBIOSIS”; more specifically, by the Custom Antibody Service (CAbS, CIBER-BBN, IQAC-CSIC). Their preparation and characterization will be described elsewhere. The tryptophan hydroxylase polyclonal antibody (ref. PA5-19749) was purchased from Thermo Fisher Scientific, (Thermo Fisher Scientific, MA, USA). A 96-well ELISA microplate was coated overnight at 4 °C with anti-KYNA and anti-TRP primary antibodies. Then, plates were washed with phosphate-buffered saline containing 0.05% Tween-20 (PBST). After washing, following standards and CSF samples (50 µL), 50 µL of HRP-bioconjugates (HRP-SIA-IHSH or HRP-L-5-HTP) were added to each well and the plate was incubated for 30 min at room temperature. Next, wells were washed and 100 µL of substrate solution were added. After 30 min incubation, absorbances were measured at 450 nm using a Fisher Multiskan Go microplate reader (Thermo Fisher Scientific, MA, USA). Inter-assay variability for KYNA and L-5-HTP assays was estimated at 21.4% (*n* = 17) and 21% (*n* = 4), respectively. Intra-assay variability was estimated at 13.4% (*n* = 18) and 10.6% (*n* = 6) for KYNA and L-5-HTP, respectively.

### 2.6. Data and Statistical Analysis

Numerical data are shown as means ± standard deviations (SD) and categorical data as a percentages. To compare demographic, clinical, and plasma and CSF biomarker data between groups, we used the non-parametric Kruskal–Wallis rank test and non-parametric pairwise comparisons adjusted by Bonferroni. Fisher’s exact test was used to compare the distribution of categorical data across groups. Correlations between biomarkers were assessed using Spearman rank correlation. Receiver operating characteristic (ROC) curves were used to determine the diagnostic accuracy of KYNA from CSF to differentiate between the diagnostic groups. The optimal cut-off point, sensitivity, specificity, and area under the curve (AUC) were calculated in all cases. Statistical significance was set out at *p* < 0.05. Statistical analysis was performed using STATA/IC 14.2 for Windows, Texas, USA.

## 3. Results

### 3.1. Demographic and Clinical Characteristics

Clinical, demographic, and genetic profiles from all subjects are shown in Table 1. No differences in sex were found between groups. Regarding age, healthy donors and ALS patients were younger than AD dementia patients. MMSE showed a progressive decreased score according to cognitive severity in the MCI and AD dementia groups. APOE genotype was more prevalent in the MCI/AD patients than in controls, according to previous publications [47]. Aβ_42_ and t-tau levels in CSF are also shown in Table 1.

### 3.2. KYNA and TRP Levels in CSF from the Study Participants

**Figure 1:**
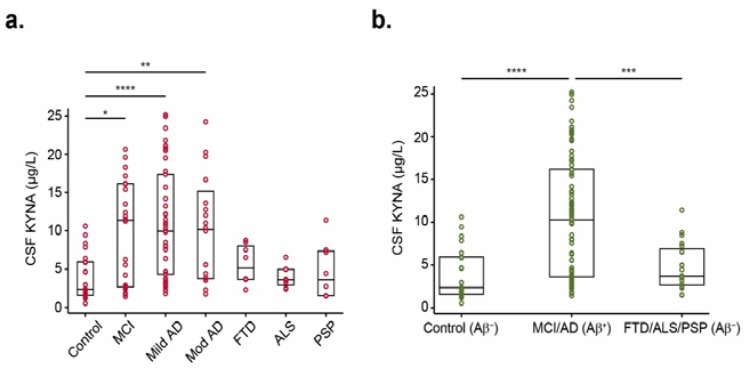
KYNA levels in CSF from all differentially diagnosed groups of donors. (**a**) Scatter plots over box plots showing KYNA levels in CSF from all groups following the diagnosis. Boxes represent the 25th, 50th, and 75th data percentiles. KYNA levels were increased in MCI and AD dementia groups compared to controls. (**b**) Scatter plots displaying KYNA levels in CSF from subjects grouped throughout their CSF biomarker profile: controls (*n* = 23), patients with AD-indicative CSF biomarker profiles (*n* = 85), and patients without AD-indicative CSF biomarker profiles (*n* = 24). Differences between groups were assessed using Kruskal–Wallis test. * *p* < 0.05; ** *p* < 0.01; *** *p* < 0.001; **** *p* < 0.0001. MCI, mild cognitive impairment; Mild AD, mild Alzheimer’s disease; Mod AD, moderate–severe Alzheimer’s disease; FTD, frontotemporal dementia; ALS, amyotrophic lateral sclerosis; PSP, progressive supranuclear palsy; Aβ^+^, AD CSF biomarker profile; Aβ^−^ non-AD CSF biomarker profile; KYNA, kynurenic acid; TRP, tryptophan.

Averaged KYNA and TRP levels in CSF from all subjects are shown in Table 1, and each KYNA individual value is represented in Figure 1. Data show increased KYNA concentrations in MCI (9.8 ± 6.9 µg/L; *p* < 0.05), mild AD (11.1 ± 7.2 µg/L; *p* < 0.0001), and moderate–severe AD (10.5 ± 7.4 µg/L; *p* < 0.01) patients compared with controls (3.9 ± 2.9 µg/L) (Figure 1a). Although the analysis of KYNA levels in other neurodegenerative diseases reported no significance when compared to MCI, mild AD, and moderate AD, a trend of reduced levels was observed in FTD (5.6 ± 2.5 µg/L), ALS (4.0 ± 1.4 µg/L), and PSP (4.7 ± 3.7 µg/L) groups. Regarding TRP levels, no differences were found between groups (Table 1). KYNA/TRP ratio was increased in CSF from AD patients compared with healthy subjects (0.06 ± 0.06), but only the mild AD group reached statistical significance (0.16 ± 0.13; *p* < 0.01; Table 1).

According to Aβ levels in CSF (Aβ^+^ or Aβ^−^ profile), KYNA levels were still found higher in MCI/AD group (Aβ^+^) compared with healthy subjects (Aβ^−^) (10.6 ± 7.1 µg/L vs. 3.9 ± 2.9 µg/L; *p* < 0.0001; Figure 1b). Additionally, it was found that KYNA levels were significantly upregulated in the MCI/AD group compared with FTD/ALS/PSP (Aβ^−^) (4.8 ± 2.7 µg/L; *p* < 0.001; Figure 1b).

To determine whether the differences of KYNA concentration in CSF between groups could be used to discriminate AD patients from healthy controls and patients with other neurodegenerative diseases, we modelled the variables using ROC curve analysis (Table 2). To differentiate MCI/AD versus healthy controls, the AUC of KYNA in CSF was 0.807 (95% CI 0.716, 0.897), while using the KYNA/TRP ratio, the AUC was 0.754 (95% CI 0.641, 0.867). We found that the optimal cut-off point for KYNA in CSF to differentiate patients with AD from healthy controls was 7.56 µg/L, giving 60% sensitivity and 82.61% specificity (Table 2). At this cut-off point, sensitivity and specificity for differentiating AD from non-AD patients were above 60% and 87%, respectively (Table 2).

### 3.3. Correlations between KYNA and TRP Levels in Plasma and CSF

A crucial topic for studies of biomarkers in AD is whether concentrations in the brain or CSF are truly reflective of concentrations of the target(s) in peripheral fluids or tissues, such as blood. To evaluate whether KP was also altered in blood, plasma levels of KYNA and TRP were determined in a patient’s subgroup with MCI (*n* = 11), mild AD (*n* = 17), moderate–severe AD (*n* = 9), and healthy controls (*n* = 20). Neither KYNA and TRP levels nor the KYNA/TRP ratio showed any significant change between patients and controls (Table 1).

**Figure 2:**
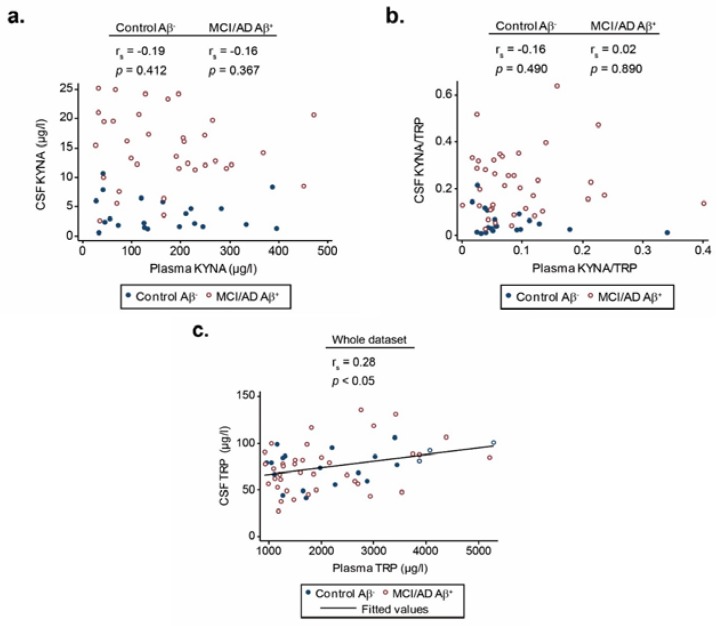
Spearman rank correlation between KYNA and TRP levels in plasma and CSF. Spearman rank correlation between KYNA (**a**), the KYNA/TRP ratio (**b**), and TRP (**c**) levels in plasma and CSF. Abbreviations: MCI, mild cognitive impairment; AD, Alzheimer’s disease; Aβ^+^, AD CSF biomarker profile; Aβ^−^ non-AD CSF biomarker profile; KYNA, kynurenic acid; TRP, tryptophan.

Plasma levels of KYNA (Figure 2a) and the KYNA/TRP ratio (Figure 2b) failed to correlate with their respective CSF concentrations. However, plasma TRP levels in the whole dataset significantly correlated with those levels in CSF (r = 0.28, *p* < 0.05; Figure 2c).

### 3.4. Correlation between KYNA Levels in CSF with Demographics and Clinical Data.

**Figure 3:**
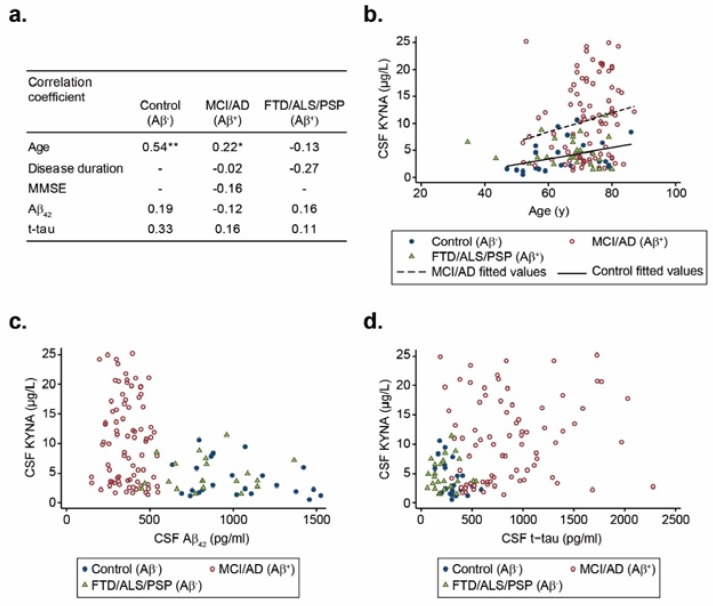
Spearman rank correlation facing KYNA levels in CSF with age, MMSE, disease duration, and Aβ_42_ and t-tau levels within the AD-biomarker-diagnostic groups. Spearman rank correlation between KYNA levels in CSF, and age (**a**,**b**), Aβ_42_ levels (**a**,**c**), and t-tau levels (**a**,**d**). Abbreviations: MCI, mild cognitive impairment; AD, Alzheimer’s disease dementia; FTD, frontotemporal dementia; ALS, amyotrophic lateral sclerosis; PSP, progressive supranuclear palsy; MMSE, Mini-Mental State Examination; Aβ_42_, amyloid-β 42; t-tau, total tau; KYNA, kynurenic acid; Aβ^+^, positive AD biomarker profile; Aβ^−^, negative AD biomarker profile. * *p* < 0.05; ** *p* < 0.01.

We investigated the correlation between KYNA levels in CSF with demographic and clinical data including Aβ_42_ and t-tau biomarkers in the participants, grouped according to their Aβ profile. There was a positive correlation with age in the control group (r = 0.54, *p* < 0.01), and MCI/AD group (r = 0.22, *p* < 0.05; Figure 3a,b), suggesting that KYNA levels in CSF increased with ageing. Regarding the disease duration and MMSE score, no correlation was found with KYNA levels in CSF. We did not find any correlation between KYNA levels with Aβ_42_ and t-tau levels in CSF either (Figure 3c,d). Our results agree with those previously reported by Jacobs and colleagues, as they did not find any significant correlations between Aβ_42_ levels in CSF and KP metabolites in samples from AD patients [34].

## 4. Discussion

The present study confirms that KYNA levels in CSF are significantly higher in MCI and AD dementia patients compared to healthy controls. We extend this result showing that such an increase seems to be specific to AD, as it is not seen in other neurodegenerative disorders, including FTD, ALS, and PSP, where KYNA levels remained unchanged.

Our findings provide evidence of an association between KYNA levels in CSF and AD pathology, in agreement with recent studies [36,37]. They are consistent with other works suggesting that increased KYNA levels in CSF from AD patients may represent an engagement of a compensatory mechanism counteracting neurotoxicity [48,49]. KYNA, described as putatively neuroprotective [18] was previously reported to be specifically associated with neuroinflammation in CNS diseases, including AD [11,29,50,51,52]. Additionally, KYNA has been shown to predict poor cognitive performance related to frontal executive functions and memory [53]. In contrast, plasma KYNA concentration remained unchanged in MCI and AD dementia patients compared to healthy subjects. This finding suggests that KYNA concentration in CSF does not depend on its systemic levels. TRP catabolism is activated by pro-inflammatory cytokines [54]. It has been shown that neural synthesis of KYNA takes place predominantly in astrocytes [55], being secreted in response to astrogliosis and inflammatory actions, both features of AD. Astrocytes are considered the primary source of extracellular KYNA concentrations [19,20]. Studies in rodents showed that kynurenine aminotransferase II (KAT II)—the enzyme that converts kynurenine to KYNA—was strongly localized in astrocytes [56,57,58]. We then may hypothesize that increased KYNA concentrations in CSF from AD patients in our cohort could reflect activated astrogliosis and increased astrocytic KAT activity, which could be part of the reactive changes that occur in astrocytes during neurodegeneration.

It is well established that neuroinflammation has a role in brains from AD affected subjects, which correlates with overproduction of kynurenine metabolites [59]. During neuroinflammation, KP catabolizes 95% of TRP pool in brain, leading to the formation of several neuroactive metabolites. This is consistent with the increased levels of KYNA reported in brains from AD patients [26]. More interestingly, raised levels of enzymes initiating TRP oxidation, indoleamine 2,3-dioxygenase 1 (IDO-1), and tryptophan 2,3-dioxygenase (TDO), were also reported in human AD brains. It has been shown that IDO-1 expression was increased in hippocampi from AD patients [59] Higher IDO-1 and TDO immunoreactivity was also observed in the hippocampi from four AD patients when compared to four age and sex-matched normal controls [60]. IDO-1 is up-regulated by certain cytokines, and inflammatory molecules such as Aβ peptides [10]. Other study showed that IDO-1 is up-regulated in the brains of AD patients, and it was found to be associated with NFT and Aβ plaques [61]. Altogether, these studies strongly support that the KP is potentially over-activated in AD, suggesting its role in the neurofibrillary tangle and senile plaque formation.

Although it was proposed that the KP is deregulated in ALS [62], scarce studies have provided direct evidence showing increased KYNA levels in CSF from ALS patients [63,64,65]. Ilzecka et al. reported that KYNA concentration in CSF was significantly higher in ALS patients with bulbar onset compared to healthy subjects, but there was no difference in KYNA concentration between the whole ALS group and healthy subjects [65].

KYNA levels in CSF may also be increased upon inflammatory conditions following immune activation. Elevated KYNA concentration in CSF was found in patients with inflammatory diseases, such as bacterial, viral, fungal, and parasitic infections; meningitis; autoimmune diseases; and septicemia, where autoimmune activation is present [34,66,67]. Recent studies on AD-associated inflammatory pathways have found an important link between infections and the immune response [68,69]. It was reported that many specific viral, bacterial, and fungal pathogens are suspected to play a role in the progression of neurodegeneration due to AD, including herpes simplex virus type 1 (HSV-1), *Chlamydia pneumonia,* spirochetes, and *Candida* [70,71]. Infection-induced inflammation triggers catabolism of TRP in several bacterial, protozoan, and viral infections, such as *Chlamydia psittaci*, *Toxoplasma gondii*, *Leishmania donovani*, and herpes simplex virus (HSV)-2 [72,73]. TRP and its catabolites are well known for their immunosuppressive functions, disease tolerance, and contributions to immune-privileged sites, such as the brain [74]. The relationship among cognitive deficits, KP mediators, and oxidative damage in the brain of an AD mouse model has been recently provided, showing an increase of TRP metabolites in the brain [75].

Among other chronic neurodegenerative disorders, a decrease in KYNA concentration was detected in brain tissue and CSF from Parkinson’s disease (PD) patients [76]. However, more recent studies reported altered levels of KP metabolites in CSF from PD patients exclusively after L-DOPA treatment [77]. Dysregulation of KP was not previously reported in FTD or PSP. The present work agrees with a recent study describing KP metabolites in CSF and plasma samples from AD patients and correlations between these metabolites and well-established AD biomarkers (t-tau and phospho-tau) [36].

## 5. Conclusions

Here we report in a new and large cohort, increased KYNA levels in CSF from AD patients compared to healthy subjects, including prodromal stages of the disease. Our data clearly demonstrate that KYNA levels in CSF are specifically higher in AD patients but not in patients with other neurodegenerative disorders. We propose that high KYNA concentration in CSF may reflect a compensatory mechanism in AD patients, based on its neuroprotective feature, but also an inflammatory response following putative immune activation. Thus, we suggest that KYNA levels in CSF have potential as a diagnostic marker of AD in combination with the existing CSF biomarkers (Aβ_42_ and tau). Along with our present findings and previous works [78,79], we suggest the KP in cerebral AD pathology to be a possible target for disease modifying interventions.

## Figures and Tables

**Table 1:** Demographic and biomarker characteristics of the study participants.

	Control	MCI	Mild AD	Mod AD	FTD	ALS	PSP	*p*
Characteristics	(*n* = 23)	(*n* = 24)	(*n* = 41)	(*n* = 20)	(*n* = 8)	(*n* = 8)	(*n* = 8)	#
Age, mean (SD), y	64.7 (10.8)	72.0 (7.1)	71.9 (8.1) ^a^	73.3 (7.2) ^a,b^	66.4 (5.2)	58.0 (14.1)	74.0 (6.0)	<0.001
Female sex, n (%)	8 (34.8)	14 (58.4)	22 (53.7)	13 (65.0)	3 (37.4)	3 (37.5)	4 (50.0)	ns
Disease duration, mean (SD), y	-	2.4 (1.4)	2.6 (1.7)	4.0 (1.6) ^c,d,e^	3.3 (1.2)	1.8 (0.9)	4.1 (2.2)	<0.01
MMSE score, mean (SD)	-	25.4 (3.2)	21.6 (3.0) ^e^	16.0 (4.3) ^f,g^	-	-	-	<0.0001
CDR, mean (SD)	0	0.5	1,0	2.2 (0.4)	-	-	-	NA
APOE ε4 carrier, No./Total (%)	0/15 (0.0%)	9/15 (60.0%) ^h^	9/18 (50.0%) ^h^	2/9 (22.2%)	-	-	-	<0.0001
**CSF biomarkers**								
Aβ_42_, mean (SD), pg/mL	1026.1 (278.9)	380.0 (89.3)	355.9 (103.8)	360.9 (88.0)	832.2 (188.5)	701.2 (224.1)	1058.7 (219.3)	NA
T-tau, mean (SD), pg/mL	303.9 (110.7)	928.8 (545.0)	836.4 (444.0)	871.2 (389.7)	305.4 (162.0)	137.0 (56.5)	252.3 (88.7)	NA
CSF KYNA, mean (SD), µg/L	3.9 (2.9)	9.8 (6.9) ^a^	11.1 (7.2) ^i^	10.5 (7.4) ^h^	5.6 (2.5)	4.0 (1.4)	4.7 (3.7)	<0.0001
CSF TRP, mean (SD), µg/L	73.7 (19.7)	75.5 (20.6)	77.1 (25.3)	79.7 (27.0)	78.4 (18.6)	67.5 (23.6)	-	ns
Ratio KYNA/TRP, mean (SD)	0.06 (0.06)	0.13 (0.09)	0.16 (0.13) ^h^	0.17 (0.17)	0.08 (0.05)	0.07 (0.04)	-	<0.05
**Plasma biomarkers**								
	(*n* = 20)	(*n* = 11)	(*n* = 17)	(*n* = 9)				
Plasma KYNA, mean (SD), µg/L	164.0 (118.5)	179.4 (126.9)	167.4 (126.4)	176.9 (82.8)	-	-	-	ns
Plasma TRP, mean (SD), µg/L	2333.3 (1216.3)	2022.7 (1335.2)	2104.7 (822.7)	1956.0 (1259.3)	-	-	-	ns
Ratio KYNA/TRP, mean (SD)	0.08 (0.07)	0.10 (0.07)	0.08 (0.09)	0.11 (0.08)	-	-	-	ns

Abbreviations: MCI, mild cognitive impairment; Mild AD, mild Alzheimer’s disease; Mod AD, moderate–severe Alzheimer’s disease; FTD, frontotemporal dementia; ALS, amyotrophic lateral sclerosis; PSP, progressive supranuclear palsy; MMSE, Mini-Mental State Examination; CDR, Clinical Dementia Rating; Aβ_42_, β-amyloid 42; t-tau, total tau; KYNA, kynurenic acid; TRP, tryptophan; NA, not applicable; ns, non-significant. # *p*-value indicates statistical difference between all groups; ^a^ *p* < 0.05 vs. control; ^b^ *p* < 0.05 vs. ALS; ^c^ *p* < 0.05 vs. MCI; ^d^ *p* < 0.05 vs. Mild AD; ^e^ *p* < 0.01 vs. ALS; ^f^ *p* < 0.001 vs. MCI; ^g^ *p* < 0.01 vs. Mild AD; ^h^ *p* < 0.01 vs. control; ^i^ *p* < 0.0001 vs. control.

**Table 2:** Diagnostic accuracy of KYNA and KYNA/TRP in CSF.

CSF KYNA				CSF KYNA/TRP			
	MCI/AD Aβ^+^ vs. Control Aβ^−^	MCI/AD Aβ^+^ vs. FTD/ALS/PSP Aβ^−^	Aβ^+^ vs. Aβ^−^		MCI/AD Aβ^+^ vs. Control Aβ^−^	MCI/AD Aβ^+^ vs. FTD/ALS/PSP Aβ^−^	Aβ^+^ vs. Aβ^−^
AUC	0.807	0.734	0.770	AUC	0.754	0.659	0.715
(95% CI)	(0.716, 0.897)	(0.639, 0.828)	(0.691, 0.848)	(95% CI)	(0.641, 0.867)	(0.543, 0.776)	(0.622, 0.802)
Sensitivity, %	60.00	60.00	60.00	Sensitivity, %	66.67	66.67	66.67
Specificity, %	82.61	87.50	85.11	Specificity, %	72.73	60.00	67.57
Accuracy, %	64.81	66.06	68.94	Accuracy, %	67.96	65.63	66.95
Cut-off, µg/L	7.56	7.56	7.56	Cut-off, µg/L	0.068	0.068	0.068
*p* #	<0.05	ns	<0.05				

Performance of KYNA (left) and ratio KYNA/TRP (right) in CSF analyzed by receiver operating characteristic (ROC) curve. **#** *p*-value indicates statistical difference between KYNA and KYNA/TRP ROC curves. Abbreviations: MCI, mild cognitive impairment; AD, Alzheimer’s disease dementia; FTD, frontotemporal dementia; ALS, amyotrophic lateral sclerosis; PSP, progressive supranuclear palsy; Aβ^+^, AD CSF biomarker profile; Aβ^−^ non-AD CSF biomarker profile; AUC, area under the curve; ns, non-significant.

## References

[1] Cornutiu G. (2015). The Epidemiological Scale of Alzheimer’s Disease. J. Clin. Med. Res..

[2] McKhann G.M., Knopman D.S., Chertkow H., Hymann B.T., Jack C.R., Kawas C.H., Klunk W.E., Koroshetz W.J., Manly J.J., Mayeux R. (2011). The diagnosis of dementia due to Alzheimer’s disease: Recommendations from the National Institute on Aging—Alzheimer’s Association workgroups on diagnostic guidelines for Alzheimer’s disease. Alzheimers Dement..

[3] Pawlowski M., Meuth S.G., Duning T. (2017). Cerebrospinal Fluid Biomarkers in Alzheimer’sDisease—From Brain Starch to Bench and Bedside. Diagnostics.

[4] Cabezas-Opazo F.A., Vergara-Pulgar K., Perez M.J., Jara C., Osorio-Fuentealba C., Quintanilla R.A. (2015). Mitochondrial Dysfunction Contributes to the Pathogenesis of Alzheimer’s Disease. Oxidative Med. Cell. Longev..

[5] Cai Q., Tammineni P. (2017). Mitochondrial aspects of synaptic dysfunction in Alzheimer’s Disease. J. Alzheimers Dis..

[6] Parameshwaran K., Dhanasekaran M., Suppiramaniam V. (2008). Amyloid beta peptides and glutamatergic synaptic dysregulation. Exp. Neurol..

[7] Wang R., Reddy P.H. (2017). Role of glutamate and NMDA receptors in Alzheimer’s Disease. J. Alzheimers Dis..

[8] Heneka M.T., Carson M.J., El Khoury J., Landreth G.E., Brosseron F., Feinstein D.L., Jacobs A.H., Wyss-Coray T., Vitorica J., Ransohoff R.M. (2015). Neuroinflammation in Alzheimer’s disease. Lancet Neurol..

[9] Sokolowski J.D., Mandell J.W. (2011). Phagocytic clearance in neurodegeneration. Am. J. Pathol..

[10] Guillemin G.J., Smythe G.A., Veas L.A., Takikawa O., Brew B.J. (2003). A beta 1-42 induces production of quinolinic acid by human macrophages and microglia. Neuroreport.

[11] Zadori D., Veres G., Szalardy L., Klivenyi P., Vecsei L. (2018). Alzheimer’s Disease: Recent concepts on the relation of mitochondrial disturbances, Excitotoxicity, Neuroinflammation, and Kynurenines. J. Alzheimers Dis..

[12] Werner-Felmayer G., Werner E.R., Fuchs D., Hausen A., Reibnegger G., Wachter H. (1989). Characteristics of interferon induced tryptophan metabolism in human cells in vitro. Biochim. Biophys. Acta.

[13] Ting K.K., Brew B., Guillemin G. (2007). The involvement of astrocytes and kynurenine pathway in Alzheimer’s disease. Neurotox. Res..

[14] Schwieler L., Larsson M.K., Skogh E., Kegel M.E., Orhan F., Abdelmoaty S., Finn A., Bhat M., Samuelsson M., Lundberg K. (2015). Increased levels of IL-6 in the cerebrospinal fluid of patients with chronic schizophrenia--significance for activation of the kynurenine pathway. J. Psychiatry Neurosci. Jpn..

[15] Mandi Y., Vecsei L. (2012). The kynurenine system and immunoregulation. J. Neural Transm..

[16] Stone T.W., Behan W.M., Jones P.A., Darlington L.G., Smith R.A. (2001). The role of kynurenines in the production of neuronal death, and the neuroprotective effect of purines. J. Alzheimers Dis..

[17] Schwarcz R., Pellicciari R. (2002). Manipulation of brain kynurenines: Glial targets, neuronal effects, and clinical opportunities. J. Pharmacol. Exp. Ther..

[18] Lovelace M.D., Varney B., Sundaram G., Lennon M.J., Lim C.K., Jacobs K., Guillemin G.J., Brew B.J. (2017). Recent evidence for an expanded role of the kynurenine pathway of tryptophan metabolism in neurological diseases. Neuropharmacology.

[19] Guillemin G.J., Kerr S.J., Smythe G.A., Smith D.G., Kapoor V., Armati P.J., Croitoru J., Brew B.J. (2001). Kynurenine pathway metabolism in human astrocytes: A paradox for neuronal protection. J. Neurochem..

[20] Kiss C., Ceresoli-Borroni G., Guidetti P., Zielke C.L., Zielke H.R., Schwarcz R. (2003). Kynurenate production by cultured human astrocytes. J. Neural Transm..

[21] Stone T.W. (2000). Development and therapeutic potential of kynurenic acid and kynurenine derivatives for neuroprotection. Trends Pharmacol. Sci..

[22] Jhamandas K.H., Boegman R.J., Beninger R.J., Miranda A.F., Lipic K.A. (2000). Excitotoxicity of quinolinic acid: Modulation by endogenous antagonists. Neurotox. Res..

[23] Hilmas C., Pereira E.F., Alkondon M., Rassoulpour A., Schwarcz R., Albuquerque E.X. (2001). The brain metabolite kynurenic acid inhibits alpha7 nicotinic receptor activity and increases non-alpha7 nicotinic receptor expression: Physiopathological implications. J. Neurosci..

[24] Stone T.W., Perkins M.N. (1981). Quinolinic acid: A potent endogenous excitant at amino acid receptors in CNS. Eur. J. Pharmacol..

[25] Braidy N., Grant R., Adams S., Brew B.J., Guillemin G.J. (2009). Mechanism for quinolinic acid cytotoxicity in human astrocytes and neurons. Neurotox. Res..

[26] Baran H., Jellinger K., Deecke L. (1999). Kynurenine metabolism in Alzheimer’s disease. J. Neural Transm..

[27] Pocivavsek A., Wu H.Q., Potter M.C., Elmer G.I., Pellicciari R., Schwarcz R. (2011). Fluctuations in endogenous kynurenic acid control hippocampal glutamate and memory. Neuropsychopharmacology.

[28] Notarangelo F.M., Pocivavsek A. (2017). Elevated kynurenine pathway metabolism during neurodevelopment: Implications for brain and behavior. Neuropharmacology.

[29] Wennström M., Nielsen H.M., Orhan F., Londos E., Minthon L., Erhardt S. (2014). Kynurenic acid levels in cerebrospinal fluid from patients with alzheimer’s disease or dementia with Lewy bodies. Int. J. Tryptophan Res..

[30] Gulaj E., Pawlak K., Bien B., Pawlak D. (2010). Kynurenine and its metabolites in Alzheimer’s disease patients. Adv. Med. Sci..

[31] Giil L.M., Midttun O., Refsum H., Ulvik A., Advani R., Smith A.D., Ueland P.M. (2017). kynurenine pathway metabolites in Alzheimer’s Disease. J. Alzheimers Dis..

[32] Schwarz M.J., Guillemin G.J., Teipel S.J., Buerger K., Hampel H. (2013). Increased 3-Hydroxykynurenine serum concentrations differentiate Alzheimer’s disease patients from controls. Eur. Arch. Psychiatry Clin. Neurosci..

[33] Widner B., Leblhuber F., Walli J., Tilz G.P., Demel U., Fuchs D. (2000). Tryptophan degradation and immune activation in Alzheimer’s disease. J. Neural Transm.

[34] Heyes M.P., Saito K., Crowley J.S., Davis L.E., Demitrack M.A., Der M., Dilling L.A., Elia J., Kruesi M.J., Lackner A. (1992). Quinolinic acid and kynurenine pathway metabolism in inflammatory and non-inflammatory neurological disease. Brain.

[35] Hartai Z., Juhász A., Rimanóczy Á., Janáky T., Donkó T., Dux L., Penke B., Tóth G.K., Janka Z., Kálmán J. (2007). Decreased serum and red blood cell kynurenic acid levels in Alzheimer’s disease. Neurochem. Int..

[36] Jacobs K.R., Lim C.K., Blennow K., Zetterberg H., Chatterjee P., Martins R.N., Brew B.J., Guillemin G.J., Lovejoy D.B. (2019). Correlation between plasma and CSF concentrations of kynurenine pathway metabolites in Alzheimer’s disease and relationship to amyloid-beta and tau. Neurobiol. Aging.

[37] van der Velpen V., Teav T., Gallart-Ayala H., Mehl F., Konz I., Clark C., Oikonomidi A., Peyratout G., Henry H., Delorenzi M. (2019). Systemic and central nervous system metabolic alterations in Alzheimer’s disease. Alzheimer Res. Ther..

[38] Sorgdrager F.J.H., Vermeiren Y., Van Faassen M., van der Ley C., Nollen E.A.A., Kema I.P., De Deyn P.P. (2019). Age- and disease-specific changes of the kynurenine pathway in Parkinson’s and Alzheimer’s disease. J. Neurochem..

[39] Duits F.H., Teunissen C.E., Bouwman F.H., Visser P.J., Mattsson N., Zetterberg H., Blennow K., Hansson O., Minthon L., Andreasen N. (2014). The cerebrospinal fluid “Alzheimer profile”: Easily said, but what does it mean?. Alzheimers Dement..

[40] Albert M.S., DeKosky S.T., Dickson D., Dubois B., Feldman H.H., Fox N.C., Gamst A., Holtzman D.M., Jagust W.J., Petersen R.C. (2011). The diagnosis of mild cognitive impairment due to Alzheimer’s disease: Recommendations from the National Institute on Aging—Alzheimer’s Association workgroups on diagnostic guidelines for Alzheimer’s disease. Alzheimers Dement..

[41] Folstein M.F., Folstein S.E., McHuhg P.R. (1975). “Mini-mental state”. A practical method for grading the cognitive state of patients for the clinician. J. Psychiatry Res..

[42] Morris J.C. (1993). The Clinical Dementia Rating (CDR): Current version and scoring rules. Neurology.

[43] Rascovsky K., Hodges J.R., Knopman D., Mendez M.F., Kramer J.H., Neuhaus J., van Swieten J.C., Seelaar H., Dopper E.G., Onyike C.U. (2011). Sensitivity of revised diagnostic criteria for the behavioural variant of frontotemporal dementia. Brain.

[44] Gorno-Tempini M.L., Hillis A.E., Weintraub S., Kertesz A., Mendez M., Cappa S.F., Ogar J.M., Rohrer J.D., Black S., Boeve B.F. (2011). Classification of primary progressive aphasia and its variants. Neurology.

[45] Brooks B.R., Miller R.G., Swash M., Munsat T.L. (2000). El Escorial revisited: Revised criteria for the diagnosis of amyotrophic lateral sclerosis. Amyotroph. Lateral Scler. Other Mot. Neuron Disord..

[46] Boxer A.L., Yu J.T., Golbe L.I., Litvan I., Lang A.E., Hoglinger G.U. (2017). Advances in progressive supranuclear palsy: New diagnostic criteria, biomarkers, and therapeutic approaches. Lancet Neurol..

[47] Heffernan A.L., Chidgey C., Peng P., Masters C.L., Roberts B.R. (2016). The neurobiology and age-related prevalence of the epsilon4 allele of Apolipoprotein E in Alzheimer’s Disease Cohorts. J. Mol. Neurosci..

[48] Schwarcz R., Bruno J.P., Muchowski P.J., Wu H.Q. (2012). Kynurenines in the mammalian brain: When physiology meets pathology. Nat. Rev. Neurosci..

[49] Anderson G., Maes M. (2014). TRYCAT pathways link peripheral inflammation, nicotine, somatization and depression in the etiology and course of Parkinson’s disease. CNS Neurol. Disord. Drug Targets.

[50] Campbell B.M., Charych E., Lee A.W., Moller T. (2014). Kynurenines in CNS disease: Regulation by inflammatory cytokines. Front. Neurosci..

[51] Braidy N., Grant R. (2017). Kynurenine pathway metabolism and neuroinflammatory disease. Neural Regen. Res..

[52] Sharma R., Razdan K., Bansal Y., Kuhad A. (2018). Rollercoaster ride of kynurenines: Steering the wheel towards neuroprotection in Alzheimer’s disease. Expert Opin. Ther. Targets.

[53] Forrest C.M., Mackay G.M., Oxford L., Millar K., Darlington L.G., Higgins M.J., Stone T.W. (2011). Kynurenine metabolism predicts cognitive function in patients following cardiac bypass and thoracic surgery. J. Neurochem..

[54] Kindler J., Lim C.K., Weickert C.S., Boerrigter D., Galletly C., Liu D., Jacobs K.R., Balzan R., Bruggemann J., O’Donnell M. (2019). Dysregulation of kynurenine metabolism is related to proinflammatory cytokines, attention, and prefrontal cortex volume in schizophrenia. Mol. Psychiatry.

[55] Maddison D.C., Giorgini F. (2015). The kynurenine pathway and neurodegenerative disease. Semin. Cell Dev. Biol..

[56] Guidetti P., Hoffman G.E., Melendez-Ferro M., Albuquerque E.X., Schwarcz R. (2007). Astrocytic localization of kynurenine aminotransferase II in the rat brain visualized by immunocytochemistry. Glia.

[57] Heredi J., Berko A.M., Jankovics F., Iwamori T., Iwamori N., Ono E., Horvath S., Kis Z., Toldi J., Vecsei L. (2017). Astrocytic and neuronal localization of kynurenine aminotransferase-2 in the adult mouse brain. Brain Struct. Funct..

[58] Song C., Clark S.M., Vaughn C.N., Nicholson J.D., Murphy K.J., Mou T.M., Schwarcz R., Hoffman G.E., Tonelli L.H. (2018). Quantitative analysis of kynurenine aminotransferase ii in the adult rat brain reveals high expression in proliferative zones and corpus callosum. Neuroscience.

[59] Guillemin G.J., Brew B.J., Noonan C.E., Takikawa O., Cullen K.M. (2005). Indoleamine 2,3 dioxygenase and quinolinic acid immunoreactivity in Alzheimer’s disease hippocampus. Neuropathol. Appl. Neurobiol..

[60] Wu W., Nicolazzo J.A., Wen L., Chung R., Stankovic R., Bao S.S., Lim C.K., Brew B.J., Cullen K.M., Guillemin G.J. (2013). Expression of tryptophan 2,3-dioxygenase and production of kynurenine pathway metabolites in triple transgenic mice and human Alzheimer’s disease brain. PLoS ONE.

[61] Bonda D.J., Mailankot M., Stone J.G., Garrett M.R., Staniszewska M., Castellani R.J., Siedlak S.L., Zhu X., Lee H.G., Perry G. (2010). Indoleamine 2,3-dioxygenase and 3-hydroxykynurenine modifications are found in the neuropathology of Alzheimer’s disease. Redox Rep. Commun. Free Radic. Res..

[62] Lee M., Guo J.P., Kennedy K., McGeer E.G., McGeer P.L. (2017). A method for diagnosing Alzheimer’s Disease based on salivary amyloid-beta protein 42 levels. J. Alzheimers Dis..

[63] Chen Y., Stankovic R., Cullen K.M., Meininger V., Garner B., Coggan S., Grant R., Brew B.J., Guillemin G.J. (2010). The kynurenine pathway and inflammation in amyotrophic lateral sclerosis. Neurotox. Res..

[64] Guillemin G.J., Meininger V., Brew B.J. (2005). Implications for the kynurenine pathway and quinolinic acid in amyotrophic lateral sclerosis. Neuro-Degener. Dis..

[65] Ilzecka J., Kocki T., Stelmasiak Z., Turski W.A. (2003). Endogenous protectant kynurenic acid in amyotrophic lateral sclerosis. Acta Neurol. Scand..

[66] Heyes M.P., Lackner A. (1990). Increased cerebrospinal fluid quinolinic acid, kynurenic acid, and L-kynurenine in acute septicemia. J. Neurochem..

[67] Heyes M.P., Brew B.J., Saito K., Quearry B.J., Price R.W., Lee K., Bhalla R.B., Der M., Markey S.P. (1992). Inter-relationships between quinolinic acid, neuroactive kynurenines, neopterin and beta 2-microglobulin in cerebrospinal fluid and serum of HIV-1-infected patients. J. Neuroimmunol..

[68] McManus R.M., Heneka M.T. (2017). Role of neuroinflammation in neurodegeneration: New insights. Alzheimers Res. Ther..

[69] Sochocka M., Zwolinska K., Leszek J. (2017). The Infectious Etiology of Alzheimer’s Disease. Curr. Neuropharmacol..

[70] Itzhaki R.F. (2014). Herpes simplex virus type 1 and Alzheimer’s disease: Increasing evidence for a major role of the virus. Front. Aging Neurosci..

[71] Itzhaki R.F., Wozniak M.A., Appelt D.M., Balin B.J. (2004). Infiltration of the brain by pathogens causes Alzheimer’s disease. Neurobiol. Aging.

[72] Adams O., Besken K., Oberdorfer C., MacKenzie C.R., Takikawa O., Daubener W. (2004). Role of indoleamine-2,3-dioxygenase in alpha/beta and gamma interferon-mediated antiviral effects against herpes simplex virus infections. J. Virol..

[73] Schmidt S.V., Schultze J.L. (2014). New insights into IDO biology in bacterial and viral infections. Front. Immunol..

[74] Moffett J.R., Namboodiri M.A. (2003). Tryptophan and the immune response. Immunol. Cell Biol..

[75] Fertan E., Rodrigues G., Wheeler R.V., Goguen D., Wong A.A., James H., Stadnyk A., Brown R.E., Weaver I.C.G. (2019). Cognitive decline, cerebral-spleen tryptophan metabolism, oxidative stress, cytokine production, and regulation of the Txnip gene in 3xTg-AD Mice. Am. J. Pathol..

[76] Ogawa T., Matson W.R., Beal M.F., Myers R.H., Bird E.D., Milbury P., Saso S. (1992). Kynurenine pathway abnormalities in Parkinson’s disease. Neurology.

[77] Havelund J.F., Andersen A.D., Binzer M., Blaabjerg M., Heegaard N.H.H., Stenager E., Faergeman N.J., Gramsbergen J.B. (2017). Changes in kynurenine pathway metabolism in Parkinson patients with L-DOPA-induced dyskinesia. J. Neurochem..

[78] Rahman A., Ting K., Cullen K.M., Braidy N., Brew B.J., Guillemin G.J. (2009). The excitotoxin quinolinic acid induces tau phosphorylation in human neurons. PLoS ONE.

[79] Kaddurah-Daouk R., Zhu H., Sharma S., Bogdanov M., Rozen S.G., Matson W., Oki N.O., Motsinger-Reif A.A., Churchill E., Lei Z. (2013). Alterations in metabolic pathways and networks in Alzheimer’s disease. Transl. Psychiatry.

